# Online and telephone access to general practice: a cross-sectional patient survey

**DOI:** 10.3399/BJGPO.2020.0179

**Published:** 2021-06-09

**Authors:** Carol Bryce, Matthew DL O'Connell, Jeremy Dale, Martin Underwood, Helen Atherton

**Affiliations:** 1 Unit of Academic Primary Care, Warwick Medical School, University of Warwick, Coventry, UK; 2 Department of Population Health and Environmental Sciences, King’s College London, London, UK; 3 Warwick Clinical Trials Unit, Warwick Medical School, University of Warwick, Coventry, UK

**Keywords:** digital health, primary health care, inequalities, internet, telephone

## Abstract

**Background:**

Improving access to primary health care in the UK has focused on the use of telephone and online access, but little is known about how awareness of and use varies between different patient groups.

**Aim:**

To determine how patients are interacting with telephone and online channels for accessing general practice services and information, and to analyse how this varies according to patient characteristics and health status.

**Design & setting:**

A cross-sectional self-administered survey of adult patients in general practices across the West Midlands, UK.

**Method:**

Descriptive statistics were used to show participants’ awareness of and interaction with online information sources and remote access. Multivariable logistic regression was used to model the relationships between demographic and health characteristics, and awareness and use of online services and alternatives to face-to-face consultations (for example, telephone).

**Results:**

A total of 2789 patients (19.0% response rate) from 43 general practices participated. The study found 60.8% (*n* = 1651/2715) of participants were aware of online services and 30.3% (*n* = 811/2674) reported having used one. Daily internet usage and frequently visiting the GP showed the strongest associations with knowledge and use of online services.

**Conclusion:**

The study shows that there is the potential for inequitable awareness and use of telephone and online services in general practice populations. Given that their use has greatly increased owing to the COVID-19 pandemic, future service design will need to ensure equity is taken into account.

## How this fits in

Evidence on how patients interact with telephone and online channels for accessing general practice services and information is lacking. The study shows that before the COVID-19 pandemic, awareness and use of telephone and online channels of access in the UK was higher in certain groups within the population. Less frequent internet use and not attending the general practice were associated with lower awareness and use of online services. When accounting for this, the study also shows differences according to factors including age, education level, and deprivation. The onset of the pandemic led to the rapid introducation of telephone and video consultation, and there remains an urgent need for strategies to avoid exacerbating existing inequalities when moving forward.

## Introduction

Communications technology is seen as a tool for improving access to primary health care.^1–3^ In the UK there has been a recent policy drive for increased use of digital services.^3,4^ However, before the COVID-19 pandemic, adoption by general practices was slow.^5,6^ At the onset of the pandemic the UK NHS rapidly implemented telephone triage and remote consultation (telephone, online, and video) in response, as a way to reduce the number of face-to-face consultations.^7^ GP face-to-face consultations dropped during March 2020 from 80.5% to 51.0%.^8^ As health services plan for the future, it is important to consider benefits^9^ and consequences that may be associated with rapid implementation of remote access.

From 2019 it was mandatory for GPs in England to provide patients with online appointment bookings, online repeat prescriptions, and access to their medical records online.^10^ Data from early 2020 show fewer than one-third of patients (29.6%) in England were registered to use any of these online services^11^ and uptake was low (ordering prescription online [18.8%], booking appointment online [18.1%], and requesting access to patient records online [5.8%]).^12^ In the UK most individuals are connected to the internet,^12^ but frequency of use and ability to use it varies greatly. Some groups may be disadvantaged by a move to online access; for example, older adults, those with a disability or long-term health condition, low socioeconomic groups, migrants, and ethnic minorities.^13^

Online and telephone access to general practice services was examined by conducting a cross-sectional survey of patients registered at general practices in the West Midlands. How patients were interacting with telephone and online channels for accessing general practice services and information was determined, and how this varied according to patient characteristics and health status was analysed. The survey now also provides baseline data before the COVID-19 pandemic.

## Method

### Study design

A cross-sectional, self-administered survey was conducted with adults registered at general practices across the West Midlands. The survey was administered between February and June 2019.

### Survey instrument

The survey had 32 questions. It was designed to collect data on demographic and health factors known to influence whether and how patients access online services and/or general practice, as well as on awareness and use of online services.^14–17^ Validated questions were used from the NHS General Practice Patient Survey^18^ (on internet usage, caring responsibilities, the responder’s experience of using their GP surgery, its website and getting an appointment, and repeat prescription) and the Office for National Statistics^19^ (on participant characteristics including age, sex, ethnic group, education level, and health status).

Questions relating to access, including knowledge and use of alternatives to a face-to-face consultation and private online providers (outside of the NHS), were devised by the study team, drawing on surveys conducted in this field and other related work.^20,21^

The survey was piloted by conducting five cognitive interviews with members of the public to check understanding, particularly in relation to the questions devised by the study team.^22^

### Sampling and recruitment

The survey was aimed to sample approximately 15 000 people. Based on estimates from previous surveys conducted in general practice, a 20% response rate was expected to be seen.^23,24^ Patients were sampled at the general practice level. It was aimed to recruit 40 practices selected to ensure the inclusion of a wide range of patients, sampling general practices purposively to ensure variation in rural and urban locations, list size, deprivation score, and proportion of patients from minority ethnic groups. Sampling of patients was proportional to general practice list size, achieved by randomly sampling 5% of eligible patients registered at each practice. Patients were excluded if they were aged <18 years, at the end of life, or lacked capacity to consent to participate in the survey. An index of multiple deprivation score was assigned to participants within each practice based on the area score for location of that practice.^25^ This scores practices from one to ten with a score of one indicating the most deprivation.^25^

### Data collection

Patients were sent a paper copy of the survey with a postage-paid return envelope. Participants were given the option to complete the survey online using a web link or a quick response (QR) code on the front of the paper survey. The online survey was administered using the software package Qualtrics. A reminder letter was sent to all sampled patients 2 weeks after the initial survey. This letter thanked them for participating (where they had) and prompted a response where they had not.

### Data analysis

Descriptive statistics were used to show participants’ awareness of and interaction with online information sources and remote access. Ethnic group was split into two categories: White; and Black, Asian, Mixed, and Other (BAMO). It was not possible to split the categories further owing to small numbers of responders from BAMO groups. Multivariable logistic regression models were used to model the relationships between demographic characteristics (age, sex, ethnic group, education level, and employment status), health characteristics (number of long-term conditions and number of visits to GP in past year), other personal characteristics (level of internet use and caring responsibilities), and awareness and use of services. The predictor variables were selected a priori based on the factors known to influence whether and how patients access online services and/or general practice that comprised the questions in the survey. Univariable models for each variable were also constructed for comparative purposes (see Supplementary Tables S1–S4). These models included clustered standard errors to account for the survey design (individuals nested within practices). Participating general practices were contacted to determine what online and telephone services they offered. General practices not offering any particular online or telephone service featured on the survey were excluded from analysis for that service. Results are expressed as odds ratios (OR) and 95% confidence intervals (95% CIs). The statistical software package Stata (version 15) was used.

## Results

In total, 43 general practices (7% of those in the West Midlands) participated; see Table 1. Of 14 694 patients sampled across the practices, 2789 (19.0%) responded, with 2413 (86.5%) returning the survey via post and 376 (13.5%) completing it online. All participating practices had a website, and offered online booking of appointments and of repeat prescriptions. Telephone appointments or callback systems were available in 40 practices, including six practices that operated a telephone triage system after all of the day appointments were booked. No practices offered email or video consultation.

**Table 1.** Practice characteristics

**Location**	**Practices, *N* =** **43, *n***
Urban	32
Rural	11
**List size (number of patients**)	
Small <6000	16
Medium 6000–12 000	20
Large >12 000	7
**Deprivation (IMD 2019**)^a^	
1–3	14
4–7	17
8–10	12
**Ethnic group, BAMO, %** ^b,c^	
<5	16
5–10	7
11–20	5
21–50	10
>50	5

^a^1 = most deprived. ^b^West Midlands BAMO population: 17.4%.^29^^c^Highest: 82.1%; lowest: 1.0%. BAMO = Black, Asian, Mixed, and Other. IMD = Index of Multiple Deprivation.

Responders’ characteristics are in Table 2. Around half of responders (51.4%; *n* = 1360/2647) reported they had a long-term physical or mental health condition, disability, or illness. Of these, 26.6% (*n* = 680/2556) reported a physical health problem, 7.9% (*n* = 201/2556) a sensory condition, 13.4% (*n* = 343/2556) a mobility problem, and 5.6% (*n* = 143/2556) a mental health problem. The majority of participants (89.0%; *n* = 2447/2748) reported their experience of general practice and of making appointments as good (71.7%; *n* = 1963/2744). Most had visited their practice at least once in the past 12 months (60.1%; *n* = 1652/2748). Patient awareness of their general practice website was high (85.0%; *n* = 2266/2666) with 42.1% (*n* = 1147/2725) having accessed the website on at least one occasion. Awareness of alternatives to face-to-face consultations as a concept was low with 0.5% (*n* = 12/2643) aware of email consultation and 0.5% (*n* = 14/2643) of video consultation.

**Table 2.** Characteristics of responders

		***n***	**%**
**Age, years,** ***n* =** **2756**	18–34	248	9.0
	35–54	680	24.7
	55–64	589	21.4
	65–74	723	26.2
	≥75	516	18.7
**Sex,** ***n* =** **2757**	Male	1181	42.8
	Female	1567	56.8
	Prefer not to say	9	0.3
**Ethnic group,** ***n* =** **2728**	White	2496	91.5
	BAMO	232	8.5
**Education,** ***n* =** **2657**	No formal qualifications	419	15.8
	Secondary level	386	14.5
	Further	588	22.1
	Higher	1115	42.0
	Still studying	18	0.7
	Other	131	4.9
**Parent or guardian for any young person aged <16 years living at home,** ***n* =** **2706**	Yes	402	14.9
	No	2304	85.1
**Caring responsibilities for family or friends,** ***n* =** **2695**	No	2112	78.4
	Yes (≤9 hours/week)	334	12.4
	Yes (≥10 hours/week)	249	9.2
**Health status: long-term physical or mental health conditions, disabilities, or illnesses,** ***n* =** **2647**	Yes	1360	51.4
	No	1287	48.6
**Frequency of using general practice services in past 12** **months,** ***n* =** **2748**	0	255	9.3
	1–5	1652	60.1
	>5	841	30.6
**Internet use in the past 3** **months,** ***n* =** **2646**	Daily	1788	67.6
	Less than daily	420	15.9
	Never	438	16.6

BAMO = Black, Asian, Mixed, and Other.

### Awareness and use of online services

Table 3 outlines the percentage of patients aware of and using different online services.

**Table 3.** Awareness and use of online services

	**Awareness, *n* =** 2715	**Use, *n* =** 2674
	***n***	**%**	***n***	**%**
**Any online service**	1651	60.8	811	30.3
**Booking appointments**	1479	54.5	552	20.6
**Ordering repeat prescriptions**	1362	50.2	584	21.8
**Accessing medical records**	629	23.2	172^a^	7.8
	**Awareness, *n*** **=** **2** **628**		**Use, *n*** **=** **2** **697**	
**Private online general practice services**	657	25.0	126	4.7
**Private online GP consultations**	451	17.2	32	1.2

a *N* = 2207.

In the multivariable model, an association between awareness of online booking for appointments and daily internet usage was observed (OR = 4.39, 95% CI = 2.98 to 6.46), higher frequency of GP visits (OR = 2.13, 95% CI = 1.41 to 3.24), having higher education qualifications (OR = 1.83, 95% CI = 1.35 to 2.48), having at least one long-term condition (OR = 1.35, 95% CI = 1.10 to 1.66), being retired (OR = 1.51, 95 % CI = 1.09 to 2.08), and being female (OR = 1.5, 95% CI = 1.24 to 1.80) (see Supplementary Table S5). Usage patterns showed the same associations with daily internet usage (OR = 14.92, 95% CI = 6.84 to 32.57), higher frequency of GP visits (OR = 10.64, 95% CI = 5.01 to 22.59), having further education qualifications (OR = 1.90, 95% CI = 1.33 to 2.73), having at least one long-term condition (OR = 1.47, 95% CI = 1.20 to 1.81), and being female (OR = 1.43, 95% CI = 1.14 to 1.79). There was also a weak association between practice deprivation score and awareness of online appointment booking with higher deprivation associated with lower awareness (OR = 1.09, 95% CI = 1.02 to 1.15), this was less clear for use (OR = 1.05, 95% CI = 0.99 to 1.12).

An association between awareness of online repeat prescriptions being offered and daily internet use was observed (OR = 5.14, 95% CI = 3.74 to 7.07), visiting the GP >5 times in the past 12 months (OR = 2.22, 95% CI = 1.56 to 3.14), having a long-term condition (OR = 1.81, 95% CI = 1.50 to 2.18), being retired (OR = 1.54, 95% CI = 1.17 to 2.03), having education qualifications (OR = 1.53, 95% CI = 1.15 to 2.03), being female (OR = 1.34, 95% CI = 1.12 to 1.61), aged 55–64 years (OR = 1.47, 95% CI = 1.03 to 2.10), and lower deprivation (OR = 1.10, 95% CI = 1.03 to 1.17) (see Supplementary Table S6). Reported use of the repeat prescription service online showed the association was strongest for those who visited the GP ≥5 times in the past 12 months (OR = 2.22, 95% CI = 1.56 to 3.14) and those who used the internet daily (OR = 11.86, 95% CI = 6.57 to 21.42). It was also significant for those with education qualifications that were not further or higher education (OR = 1.79, 95% CI = 1.10 to 2.90), having an informal caring role of ≥10 hours per week (OR = 1.51, 95% CI = 1.08 to 2.11), being retired (OR = 1.55, 95% CI = 1.11 to 2.16), and lower deprivation (OR = 1.11, 95% CI = 1.02 to 1.21).

Awareness of online access to medical records was associated with using the internet every day (OR = 4.20, 95% CI = 2.90 to 6.09), visiting the GP >5 times in the past 12 months (OR = 3.29, 95% CI = 1.80 to 6.03), being older (aged 65–74 years) (OR = 1.77, 95% CI = 1.24 to 2.52), having a long-term condition (OR = 1.38, 95% CI = 1.12 to 1.70), being retired (OR = 1.40, 95% CI = 1.06 to 1.85), and lower deprivation (OR = 1.18, 95% CI = 1.07 to 1.30). Being from a non-White ethnic group was also associated with awareness (OR = 2.05, 95% CI = 1.35 to 3.11) (see Supplementary Table S7). Access was more strongly associated with use of the internet (OR = 8.00, 95% CI = 2.63 to 24.30), frequently visiting the GP (OR = 19.88, 95% CI = 2.50 to 157.87), and having a long-term condition (OR = 2.21, 95% CI = 1.34 to 3.63).

### Awareness and use of telephone consultation

Telephone consultations were widely offered as an alternative to face-to-face consultations. Overall, 55.7% of responders (*n* = 1471/2643) were aware of telephone consultation and 36.7% (*n* = 987/2691) had used the service. Awareness of telephone consultation was associated with being female (OR = 1.82, 95% CI = 1.48 to 2.23), frequent internet use (OR = 1.81, 95% CI = 1.27 to 2.59), visiting the GP >5 times in the past 12 months (OR = 2.74, 95% CI = 1.76 to 4.24), having a long-term condition (OR = 1.40, 95% CI = 1.12 to 1.75), being a parent of a child aged <16 years (OR = 1.44, 95% CI = 1.08 to 1.93), and having an informal caring role of ≥10 hours per week (OR = 1.54, 95% CI = 1.08 to 2.20) (see Supplementary Table S8). Reported use of telephone consultations followed the same pattern, with higher education (OR = 1.51, 95% CI = 1.04 to 2.20) also being associated with use of telephone consultation.

## Discussion

### Summary

The study shows that before the pandemic, in the study population, awareness and use of online primary care services was higher in individuals who used the internet daily, and those who attended general practice frequently. Having a long-term condition, being female, and registered at a practice in an area of low deprivation were all associated with greater awareness and use of telephone and online services. Also, the study has demonstrated awareness and use of telephone and online access being associated with being retired and with higher education levels. Parents and carers were most likely to use telephone access. It was observed that awareness of the practice website and online services was higher than usage.

### Strengths and limitations

The survey included a diverse range of practices. A 19.0% patient response rate is consistent with unsolicited community postal surveys.^26^ However, it is likely that those who responded were more interested in the topic and may have differed in other ways to the non-responders; therefore, their responses cannot necessarily be extrapolated to the entire community.

Responders were older than the resident population in the West Midlands (aged ≥65 years, survey: 45.1%; West Midlands: 18.6%^27^). However, apart from high consultation rates in infants, consultation rates increased with increasing age from the lowest levels in the 15–24 age group.^6^ Consultation rates are also higher in areas of high deprivation,^28^ and, in this study, deprivation score was only available at the practice level that lacked granularity. This means the authors cannot be confident that they engaged a wide enough range of patients across the deprivation gradient and that in controlling for deprivation in the models they used only an approximation for deprivation level. The views of ethnic minority groups are also under-represented in the findings, with the proportion of responders from these groups lower than in the general population (White ethnic group, this survey: 91.5%; West Midlands region: 82.7%^29^). The findings of this study should be interpreted in light of these limitations.

The survey tool used validated questions from both the Office for National Statistics and the NHS General Practice Patient Survey, alongside questions developed to address the topic of access to general practice. Although additional validation of the survey as a whole was not conducted, cognitive interviews were used with public contributors to check understanding of the survey instrument before it was used in the study population.

Practices were sampled purposively and consequently practice level variables were not included, other than deprivation (as a proxy for patient deprivation level), in the models. Clustered standard errors were included to account for the nesting of participants within practices. Practice level factors, such as location (rural versus urban), often influence patient behaviour in general practice and so any future surveys should consider these practice level variables.

### Comparison with existing literature

The data found similar results to the national General Practice Patient Survey by NHS England,^11^ with 89.0% of the responders and 83% of the national survey rating overall experience of their general practice as good, and 71.7% of the present study's sample compared with 67% of the national sample rating their experience of making an appointment as good. This suggests the participants are broadly similar in views, despite the sample in the present study being smaller and skewed towards older people.

It was found that online services, including online booking, repeat prescription ordering, and access to patient records, were available at the majority of the general practices at the time of the survey, but uptake was limited. This is in line with national data that shows in January 2020 less than one-third of patients in England (29.6%) were registered for at least one online service.^30,31^ By 31 December 2020, the number had risen to 32.4%,^30^ a modest increase given that the majority of contacts with UK general practices became remote during the COVID-19 pandemic.^32^ Previous research shows that uptake of online or digital services remain low owing to a lack of awareness or engagement within practice populations alongside staff reservations and understanding about appropriate use.^33,34^ It is also known that deprivation has an impact on service quality, satisfaction, and usage.^35,36^

### Implications for research and practice

The COVID-19 pandemic markedly increased levels of use of telephone consultation and online consultation, and it looks likely that the rapid move to using online methods of contact and access will be retained after the pandemic to a certain degree.^7,9^ The sample was comprised of mostly older participants from a White ethnic group, who were retired. It is likely that awareness and use of online and telephone services is even lower in groups known to be disadvantaged in relation to accessing services; for example, people who use the internet less frequently, those who visit the general practice infrequently, older people, those with lower levels of education, and those who live in areas of high deprivation. Practices should raise awareness of services available, being cautious to avoid assumptions over how patients get their information. Awareness of the general practice website was high, but less than half of the sample had used it, so this is unlikely to provide a primary information source for patients about what services are available, nor does information provision alone lead to use. Patients may require support to both learn about and use services that provide alternative routes of access.

Patients who regularly attend the practice differ in relation to awareness and use of online services relative to those who attend less frequently if at all, and so different approaches are likely to be needed depending on the patient.

The burden of COVID-19 ill health and economic disadvantage is not equally distributed across communities,^37–39^ and may be hitting hardest where awareness and use of online and telephone access is lowest, risking inequality. Future research studies should explore the role of the practice, as well as patient characteristics, in determining awareness and use of services. They should also aim to produce practical recommendations about what can be done in everyday practice to ensure patients have a suitable route of access available to them.
